# Tendon stem/progenitor cells are promising reparative cell sources for multiple musculoskeletal injuries of concomitant articular cartilage lesions associated with ligament injuries

**DOI:** 10.1186/s13018-023-04313-3

**Published:** 2023-11-15

**Authors:** Heyong Yin, Kelei Mao, Yufu Huang, Ai Guo, Lin Shi

**Affiliations:** Department of Orthopaedics, Beijing Friendship Hospital, Capital Medical University, Beijing, 100053 China

**Keywords:** Cartilage, Ligament, Associated lesions, Tendon stem/progenitor cell, Cartilage repair

## Abstract

**Background:**

Trauma-related articular cartilage lesions usually occur in conjunction with ligament injuries. Torn ligaments are frequently reconstructed with tendon autograft and has been proven to achieve satisfactory clinical outcomes. However, treatments for the concomitant articular cartilage lesions are still very insufficient. The current study was aimed to evaluate whether stem cells derived from tendon tissue can be considered as an alternative reparative cell source for cartilage repair.

**Methods:**

Primary human tendon stem/progenitor cells (hTSPCs) were isolated from 4 male patients (32 ± 8 years) who underwent ACL reconstruction surgery with autologous semitendinosus and gracilis tendons. The excessive tendon tissue after graft preparation was processed for primary cell isolation with an enzyme digestion protocol. Decellularization cartilage matrix (DCM) was used to provide a chondrogenic microenvironment for hTSPCs. Cell viability, cell morphology on the DCM, as well as their chondrogenic differentiation were evaluated.

**Results:**

DAPI staining and DNA quantitative analysis (61.47 μg per mg dry weight before and 2.64 μg/mg after decellularization) showed that most of the cells in the cartilage lacuna were removed after decellularization process. Whilst, the basic structure of the cartilage tissue was preserved and the main ECM components, collagen type II and sGAG were retained after decellularization, which were revealed by DMMB assay and histology. Live/dead staining and proliferative assay demonstrated that DCM supported attachment, survival and proliferation of hTSPCs with an excellent biocompatibility. Furthermore, gene expression analysis indicated that chondrogenic differentiation of hTSPC was induced by the DCM microenvironment, with upregulation of chondrogenesis-related marker genes, COL 2 and SOX9, without the use of exogenous growth factors.

**Conclusion:**

DCM supported hTSPCs attachment and proliferation with high biocompatibility. Moreover, TSPCs underwent a distinct chondrogenesis after the induction of a chondrogenic microenvironment provided by DCM. These results indicated that TSPCs are promising reparative cell sources for promoting cartilage repair. Particularly, in the cohort that articular cartilage lesions occur in conjunction with ligament injuries, autologous TSPCs can be isolated from a portion of the tendon autograph harvested for ligaments reconstruction. In future clinical practice, combined ligament reconstruction with TSPCs- based therapy for articular cartilage repair can to be considered to achieve superior repair of these associated injuries, in which autologous TSPCs can be isolated from a portion of the tendon autograph harvested for ligaments reconstruction.

## Introduction

Along with the high participation in sports activities, trauma-related musculoskeletal injuries including ligament tear and articular cartilage lesion are prevailing in orthopedic surgery. Articular cartilage lesions usually occur in conjunction with ligament injuries [1]. It has been reported that articular cartilage lesions were found in 17–43% of the patients with anterior cruciate ligament (ACL) tears [2, 3]. For complete ligament tear, standard surgical procedure is to reconstruct the torn ligament with autologous tendon autografts, such as hamstring tendon, patellar tendon, and peroneus longus tendon and demonstrates satisfactory clinical outcomes in most of the patients [4]. However, the current therapy options such as microfracture, osteochondral autograft, and autologous chondrocyte implantation (ACI) can hardly achieve functional hyaline cartilage regeneration in long-term clinical follow-up [5, 6]. In recent years, stem cell-based regenerative strategies have emerged as promising alternative treatment for facilitating cartilage repair and regeneration [7].

Mesenchymal stem cells (MSCs) derived from bone marrow (BMSCs) and adipose tissue (ASCs) are the most widely used cell sources for cartilage regeneration, due to their easy accessibility and strong potential towards chondrogenic differentiation [8–11]. However, their applications in vivo were frequently associated with ectopic ossification and adipose tissue accumulation [12]. Tendon tissue also harbor a group of a multipotent cell, namely TSPC [13–15]. Tan et al. showed that TSPCs had higher clonogenicity and proliferative capacity, and greater chondrogenic differentiation potential than BMSCs [16]. In addition, Stanco et al. demonstrated that TSPCs have a stronger ability towards chondrogenic differentiation than ASCs [17]. These researches supported that TSPCs might be better MSC source for musculoskeletal repair and regeneration. Particularly, in the cohort that articular cartilage lesions occur in conjunction with ligament injuries, autologous TSPCs can be isolated from a portion of the tendon autograft harvested for ligaments reconstruction.

The microenvironment surrounding stem cells plays a key role in governing cell survival, proliferation and differentiation [18–21]. Because of their good biocompatibility and natural origin, DCM has been considered as an ideal biomaterial and was widely used in the field of cartilage tissue engineering [5, 22, 23]. However, no study has reported the influence of DCM on the biological behaviors of TSPCs yet.

Based on above, the current study aimed to provide a chondrogenic microenvironment for hTSPCs with DCM. We hypothesized that DCM would not only have good biocompatibility, but also induce chondrogenesis of hTSPCs, thus indicating TSPCs isolated from tendon autograft can be applied to enhance musculoskeletal repair in concomitant articular cartilage lesions associated with ligament injuries.

## Methods

### Cell isolation and expansion

Human TSPCs were isolated from 4 patients who underwent ACL reconstruction surgery with autologous semitendinosus and gracilis tendons. The main portion of the harvested tendons were used for the graft, and the excessive tendon tissue was processed for primary cell isolation. All the donors were young male with an average age of 32 ± 8 years. All the tissue harvest and cell isolation protocols were conducted with informed consent and approved by the Ethics Committee. According to the previously established procedure [24], the harvested tendon was minced and digested fully in 0.15% collagenase II (Solarbio, Beijing, China) at 37 °C overnight. The digested cells were collected by centrifuge and cell suspensions were cultured in DMEM/F-12 (Gibco, New York, USA) supplemented with 10% fetal bovine serum (Gibco, New York, USA), 1% ascorbic acid I (STEMCELL Technologies, Vancouver, Canada), 1% MEM amino acids I (Gibco, New York, USA) and 100 U/ml penicillin/streptomycin (Procell, Wuhan, China). At 80–90% confluence, cells were trypsinized, centrifuged, resuspended in growth medium for passaging. Cells at passages 3 to 6 were used in subsequent experiments.

### Preparation of decellularization cartilage matrix

In this study, fresh porcine knee femoral articular cartilage slices were obtained from a local slaughterhouse. Subsequently, the specimens were thoroughly rinsed with phosphate-buffered saline (PBS) and decellularized as previously reported [5]. Briefly, the fresh porcine knee femoral articular cartilage slices were treated with 5 freeze–thaw cycles (one cycle: freezing for 1 min in liquid nitrogen followed by thawing for 5 min in a water bath at 37 °C). Afterwards, cartilage slices were treated with 1% SDS (Sigma-Aldrich) for 8 h. After washing in PBS for 30 min, samples were digested in 50 U/mL DNase I solution (Sigma-Aldrich) at 37 °C for 4 h, followed by washing 6 times with PBS for 30 min. And then, the decellularization cartilage matrix was cut into 10-μm- thick slices by using a frozen sectioning machine. The 10-μm- thick DCM was stored in 4 °C for subsequent cell culture experiments.

### Histological and immunohistochemical evaluation

Cartilage slices were fixed with 10% neutral buffered formalin, dehydrated through a series of ascending ethanol concentrations, embedded in paraffin, and finally, longitudinal sections of 10 μm were mounted on glass slides for histological analysis. DAPI staining was performed to verify the decellularization of DCM. Hematoxylin and eosin (H&E) staining was used to characterize the general structure, nuclei, and cellular content of the matrices. Alcian blue staining was conducted to visualize the glucosaminoglycan (sGAG) content within DCM. For immunohistochemical analysis, cartilage slices were incubated with primary antibody against COL1 (Abcam, ab270993, 1:200) overnight at 4 °C. Next day, specimens were treated with secondary antibody (Abcam) for 1 h at room temperature, and then collagen I deposition was visualized by the DAB (Servicebio, Wuhan, China).

### Biochemical assays

The DNA content was quantified using the QuantiFluor® double-standred DNA (dsDNA) kit (Promega Corporation, Madison, WI, USA), according to the manufacturer’s instructions. Briefly, samples were freeze-dried with constant weight and digested in papain solution in a water bath for 24 h at 60 °C. Finally, the solution was centrifuged at 10,000 g for 30 min, and the papain digestion solution obtained was assayed using the kits. In addition, an aliquot of the digestion solution was assayed for sGAG content using the dimethyl methylene blue (DMMB) dye binding assay (Sigma) according to the manufacturer’s instruction.

### Evaluation of DCM biocompatibility and TSPCs proliferative activity

To understand the effect of DCM on cell survival and proliferation, hTSPCs were cultured on the surface of DCM slices. Live/dead staining and cell proliferation assay CCK-8 were conducted at 3, 5, and 7 days. The viability of hTSPCs cultured in the DCM were assessed using the Calcein/PI Cell Viability/Cytotoxicity Assay Kit (C2015S, Beyotime, China) according to the manufacturer’s instructions. Briefly, sterilized DCM were placed in a 48-well plate and hTSPCs were seeded on the surface of DCM with a cell density of 1 × 10^5^ cells/ml. At each time point, samples were washed with PBS and incubated in the dilute dye solution for 30 min at room temperature. After washing with PBS, these samples were observed and photographed using a laser scanning confocal microscope (LSCM, Zeiss LSM710, Carl Zeiss, Germany). For cell proliferation assay, a working solution composed of culture medium and CCK-8 reagent (9:1) was added to each well followed by incubation at 37 °C for 1 h. The optical density (OD) values of the working solution (*n* = 5) were measured at 450 nm using a microplate reader (Beckman, Fullerton, CA).

### Visualization of TSPC morphology on DCM

Phalloidin-based F-actin staining was performed to visualize morphology of hTSPC cultured on the surface of the DCM. After 3-, 5-, and 7-days cultivation, samples were fixed with 4% paraformaldehyde for 15 min, permeabilized with 0.1% Triton X-100 for 5 min, blocked with 1% BSA for 1 h, stained with Phalloidin for 30 min and counterstained with DAPI for 5 min at room temperature under dark conditions. The stained samples were observed using LSCM (LSCM, Zeiss LSM710, Carl Zeiss, Germany).

### Scanning electron microscopy

For scanning electron microscopy (SEM) observation, the samples were washed twice with PBS and fixed with 4% paraformaldehyde for 1 h. They were then dehydrated in ethanol solution at concentrations of 50%, 70%, 80%, 90%, and 100% and subjected to critical point drying. After sputtering with gold, the samples were visualized under SEM (Model S-4800, Hitachi Co. Ltd., Japan) at 5 kV.

### Quantitative real-time reverse transcription-polymerase chain reaction (qRT-PCR)

After hTSPCs were cultured on the surface of DCM for 3, 5, and 7 days, total RNA was extracted from the cells using Trizol reagent (Invitrogen) and reverse transcribed from RNA to cDNA using ReverTra Ace qPCR RT kit (Toyobo, Osaka, Japan) according to the manufacturer's protocol. Reactions were performed at 95 °C for 5 min, then 15 s at 95 °C, 15 s at 58 °C, and 45 s at 72 °C for 40 cycles. The 2^−△△Ct^ method was used to analyze RT-PCR results and GAPDH was used as a housekeeping gene. The primer sequences used for RT-PCR are as follows. SOX9 (forward: 5′-GCGGAGGAAGTCGGTGAAGAAT-3′, reverse: 5′AAGATGGCGTTGGGCGAGAT-3′), Aggrecan (forward: 5′-GGAGGAGCAGGAGTTTGTCAA-3′, reverse: 5′-TGTCCATCCGACCAGCGAAA-3′), Collagen type I (forward: 5’-GCCACCTGCCAGTCTTTACA-3′, reverse: 5′-CCATCATCACCATCTCTGCCT-3′), Collagen type II (forward: 5′-CACGCTCAAGTCCCTCAACA-3′, reverse: 5′-TCTATCCAGTAGTCACCGCTCT-3′), OCT-4 (forward: 5′-CTCGAGAAGGATGTGGTCCG-3′, reverse: 5′-TAGTCGCTGCTTGATCGCTT-3′), Nanog (forward: 5′-CAATGGTGTGACGCAGAAGG-3′, reverse: 5′-GCATCCCTGGTGGTAGGAAG-3′). GAPDH (forward: 5′-CAAGAAGGTGGTGAAGCAGG-3′, reverse: 5′-CACTGTTGAAGTCGCAGGAG-3′).

### Statistical analysis

All data were expressed as mean ± standard deviation. Student’s t-test was used to determine significant differences between two groups. ANOVA was used to determine statistical significant between multiple groups. Statistical analyses were performed using SPSS 17.0 software (SPSS, Inc., Chicago, IL, USA), and *p* < 0.05 indicated statistical significance.

## Results

### Evaluation of the decellularized cartilage matrix

**Fig. 1:**
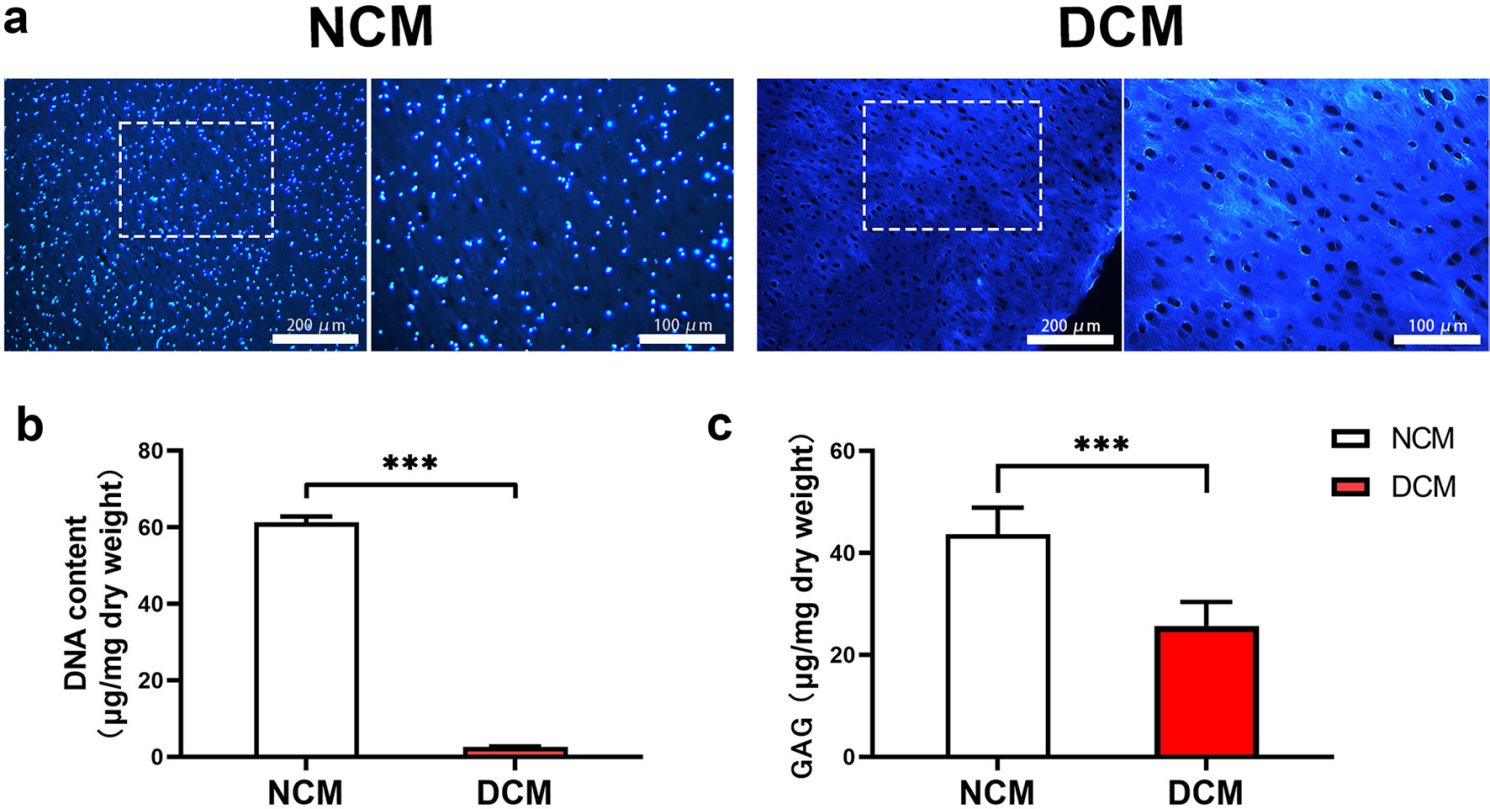
Evaluation of cartilage matrix decellularization. **a** Representative DAPI staining of the NCM and DCM. The white rectangle indicates the area shown on the right images at higher magnification. **b** Quantitative DNA content before and after the decellularization process (*n* = 4). Bar charts present mean ± standard deviation; ***p < 0.0001. **c** Quantitative GAG content before and after the decellularization process (*n* = 4). Bar charts present mean ± standard deviation; ***p < 0.0001. NCM: native cartilage matrix; DCM: decellularized cartilage matrix

**Fig. 2:**
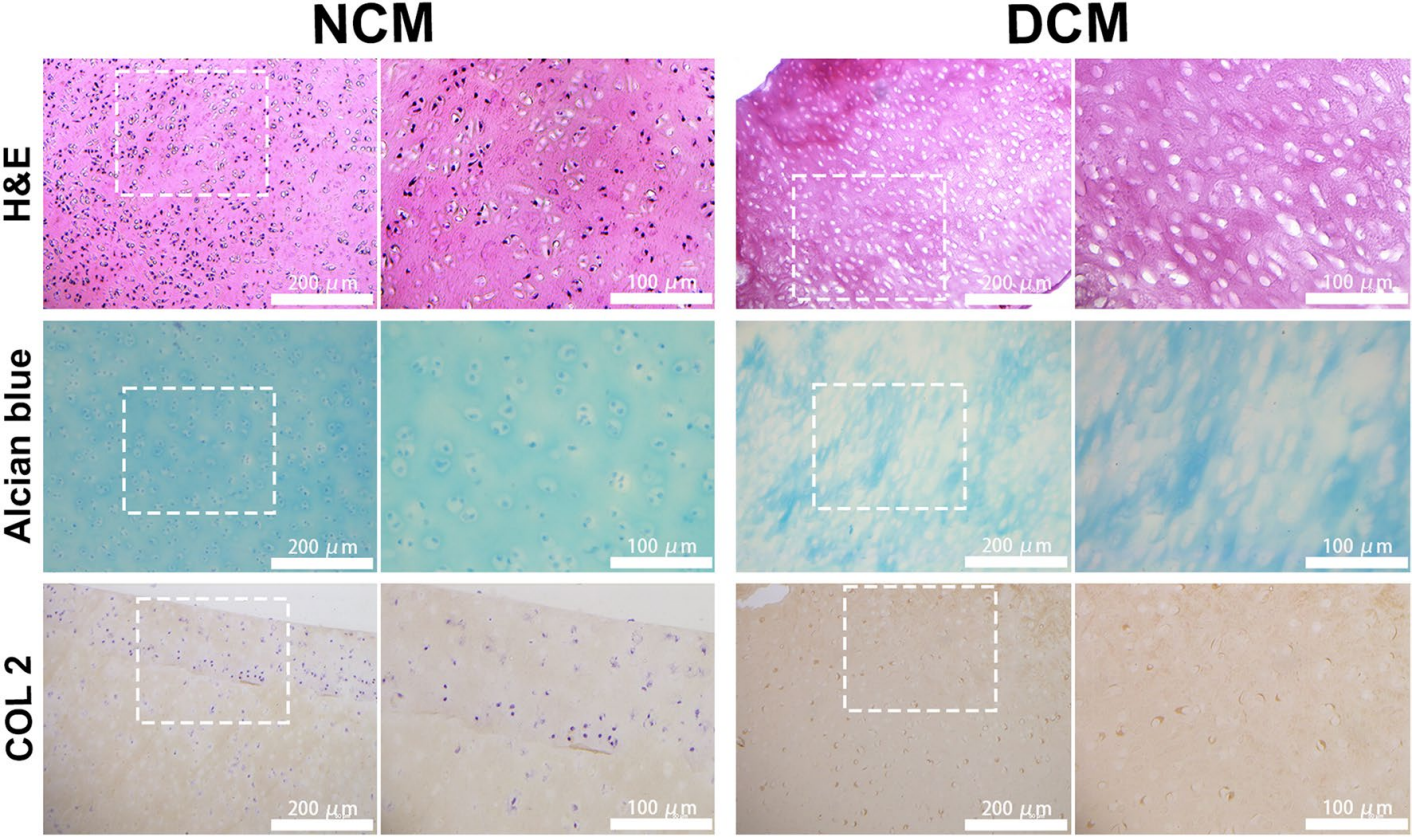
Histological evaluation of cartilage matrix before and after decellularization by H&E staining, Alcian blue staining, and immunohistochemical staining against COL2. The white rectangle indicates the area shown on the right images at higher magnification. NCM: native cartilage matrix; DCM: decellularization cartilage matrix

DCM slices were prepared via freeze–thaw cycles and chemical decellularization procedures. Decellularization aims to substantially remove the cellular components, whereas maximally preserve the ECM of the cartilage matrix samples. Specifically, for the decellularization of DCM, the clearance of the chondrocyte components was demonstrated by DAPI staining, DNA content assay, and H&E staining. Notably, the results of DAPI and HE staining demonstrate the chondrocytes present in the cartilage lacuna were almost removed after decellularization, while the basic structure of the cartilage tissue was preserved (Fig. 1). The retention of the main cartilage ECM components, collagen type II and sGAG were demonstrated by Alcian blue staining, immunohistochemical staining of COL2 and GAG content DMMB assay. Alican blue staining showed that most of the GAGs were retained within the DCM after decellularization (Fig. 2). Next, immunohistochemical staining of COL2 demonstrated that no distinct change in staining intensity of collagen before and after decellularization process (Fig. 2). Furthermore, it was confirmed by DNA assay, which shows that the average DNA content remaining in DCM was 2.64 μg per mg dry weight of the total ECM, in contrast to 61.47 μg/mg that remained in the samples before decellularization (Fig. 1b). The quantification of GAG content before and after decellularization was verified by DMMB assay, which indicated that the remaining GAG in DCM were averagely about 25.66 μg/mg dry weight, in contrast to 46.28 μg/mg before decellularization (Fig. 1c).

### Cell viability and proliferation on the surface of the DCM

**Fig. 3:**
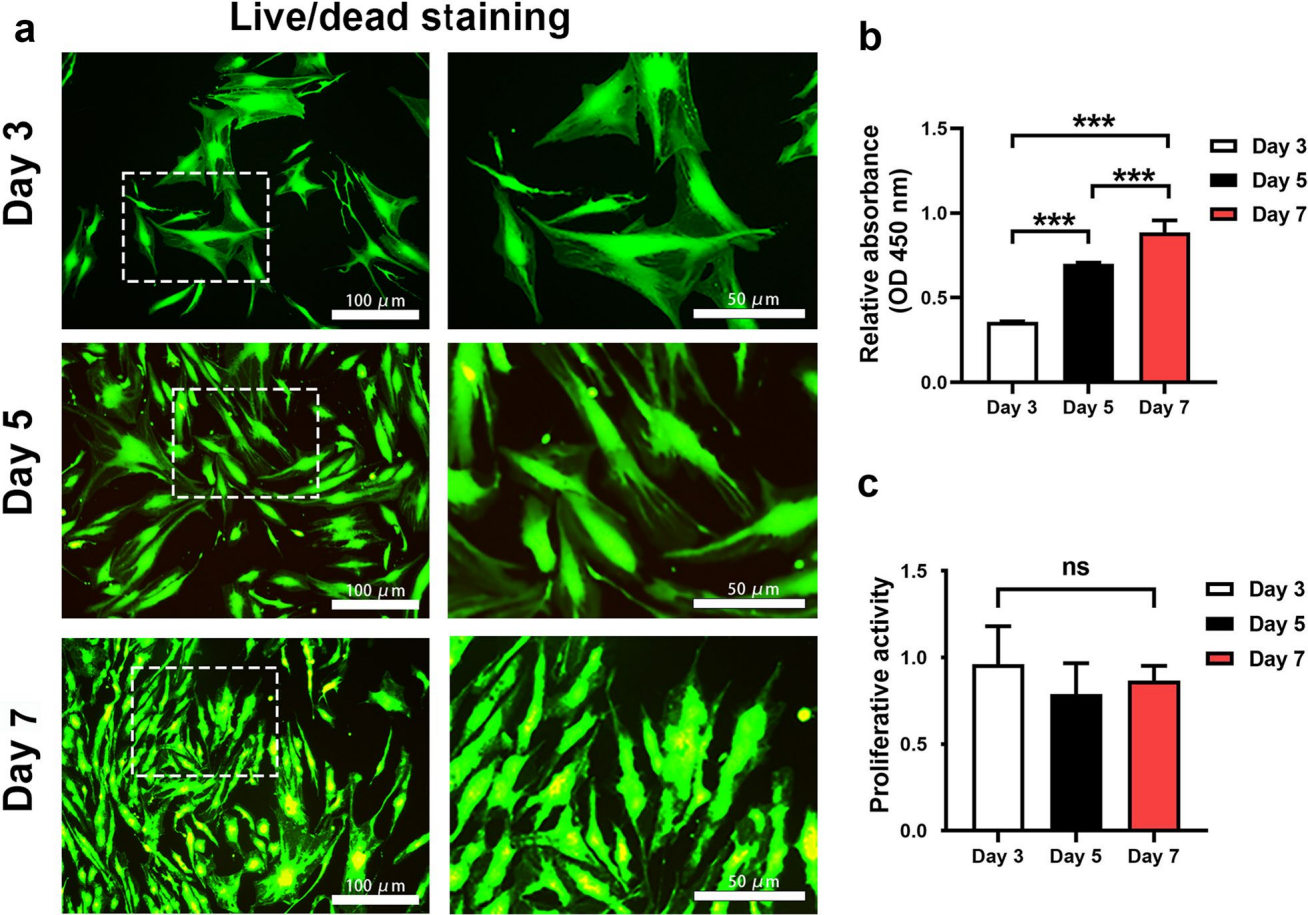
Investigation of cell survival and proliferation on the DCM. **a** Representative live/dead staining of hTSPCs loaded on the DCM at day 3, 5, and 7. The white rectangle indicates the area shown on the right images at higher magnification. **b** CCK-8 assay evaluating hTSPCs proliferation loaded on the DCM at day 3, 5, and 7 (*n* = 8). Bar charts present mean ± standard deviation; ***p < 0.0001. **c** The cell relative proliferation activity of TSPCs loaded on DCM with that on the cell culture flask (*n* = 8). ns, no significance. NCM: native cartilage matrix; DCM: decellularized cartilage matrix

Biocompatibility of the DCM and cell proliferation on the DCM slices were examined at different time points by live/dead staining (live and cells were fluorescently labeled green and dead cells were red) and CCK-8 assay. Live/dead staining from 3 to 7 days indicated that a high percentage of live hTSPCs and very few dead cells were visible on the surface of DCM (Fig. 3a). The proliferative activity of the hTSPCs loaded on the DCM was further verified by the CCK-8 at day 3, 5, and 7, which indicated that hTSPCs significantly expanded over the culture time (Fig. 3b). In addition, no significant difference of proliferative activity of hTSPCs on the surface of DCM and two-dimensional cell culture flask was detected (Fig. 3c).

### Assessment of cell morphology on the surface of the DCM

**Fig. 4:**
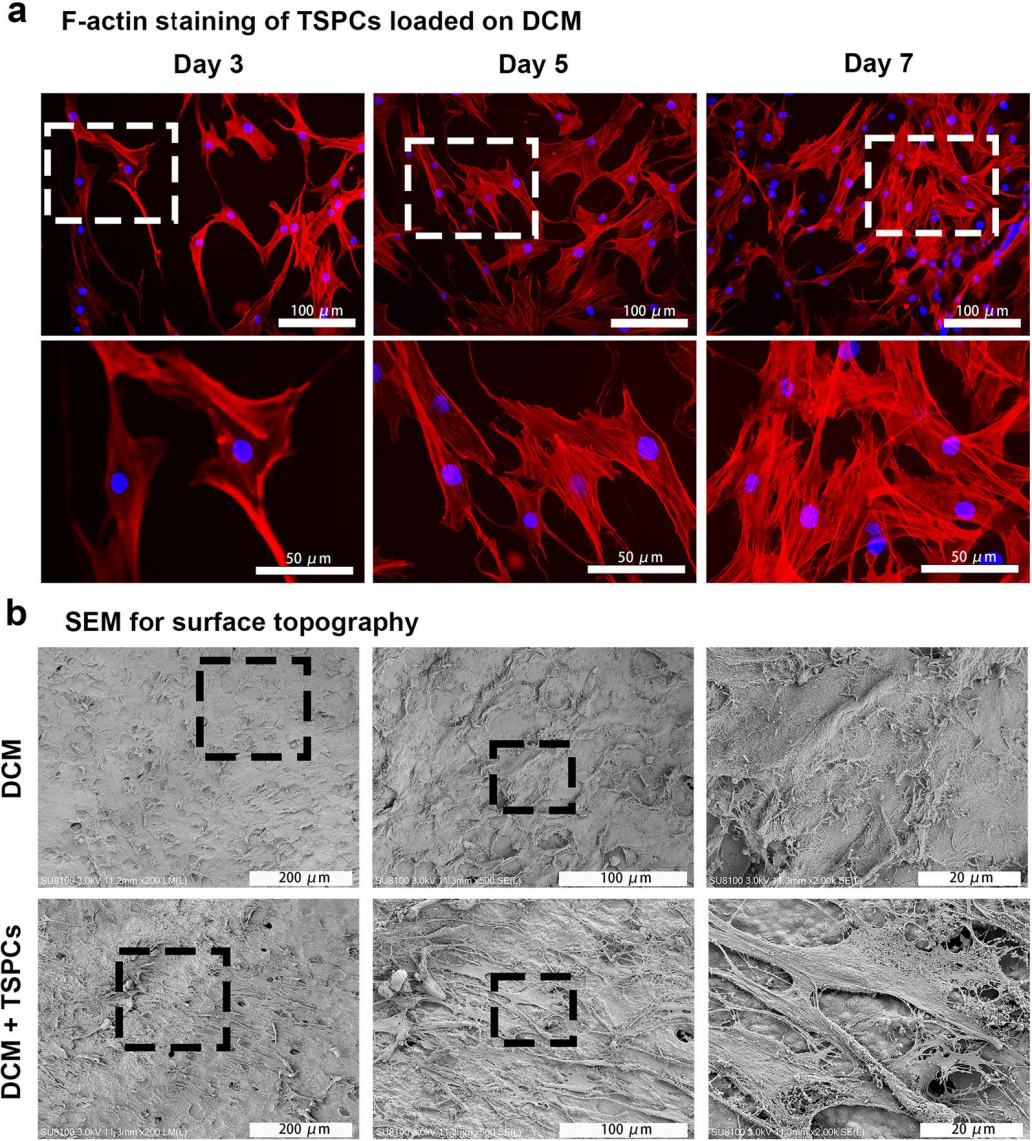
Cell morphology analysis of hTSPCs on the DCM. **a** Representative images of Phalloidin-based F-actin staining of hTSPCs grown on DCM on days 3, 5 and 7. The white rectangle indicates the area shown on the right images at higher magnification. **b** The surface topography of the DCM and the cell morphology of hTSPCs loading on DCM at day 3 were determined by SEM. The black rectangle indicates the area shown on the right images at higher magnification. NCM: native cartilage matrix; DCM: decellularized cartilage matrix

To reveal the influence of the DCM on cell morphology and cytoskeleton organization, phalloidin-based F-actin staining was performed. It can be seen that the cytoskeleton of hTSPCs loaded on the DCM is attached onto the DCM surface in a spindle-shaped pattern at day 3 (Fig. 4a). With increasing culture time, the hTSPCs grown on the DCM gradually tended to be a polygonal or even round shape at day 5 and 7 (Fig. 4a). SEM results revealed that the DCM retains the structure of cartilage lacuna and provided a smooth topography for cell attachment (Fig. 4b). After seeding on the DCM, hTSPCs attached and secreted matrix onto the surface of the DCM (Fig. 4b).

### Chondrogenic differentiation of hTSPCs loaded on DCM

**Fig.5:**
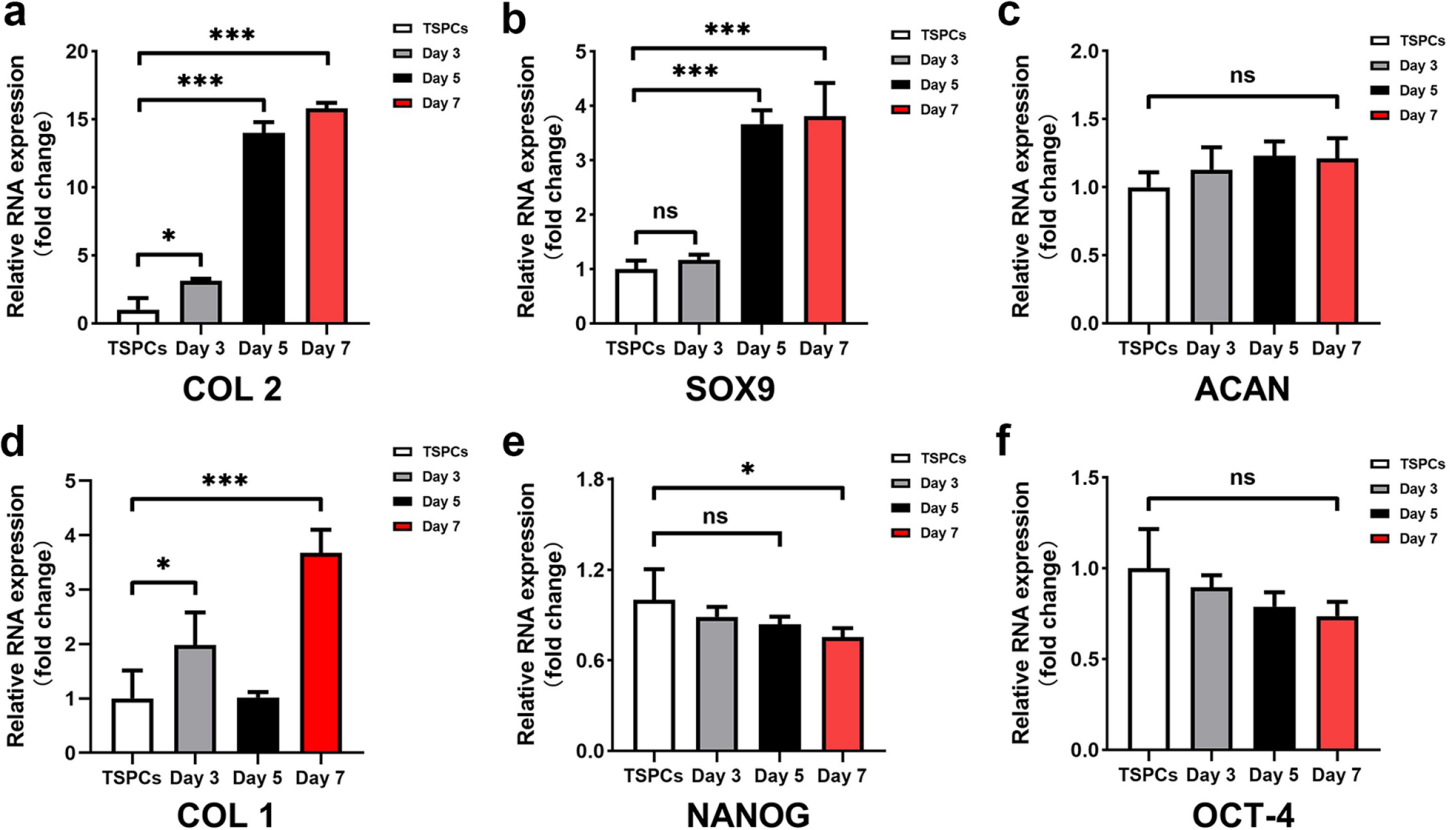
Gene expression of hTSPCs loaded on the DCM. Quantitative gene expression analysis of cartilage-related COL2, SOX9, and ACAN (**a**–**c**), COL1 (**d**) and stemness-related NANOG and OCT-4 (**e**, **f**) gene expression of hTSPCs loaded on the DCM at day 3, 5, and 7 (*n* =). Bar charts present mean ± standard deviation; *p < 0.05, ***p < 0.0001, ns, no significance. The gene expression levels are normalized with the housekeeping gene, GAPDH

Gene expression of hTSPCs cultured on DCM at day 3, 5 and 7 was quantified at mRNA expression level by RT-qPCR. The mRNA expression levels of COL1, chondrogenic marker genes (COL2, SOX9, and Aggrecan), and stemness marker genes (OCT-4 and NANOG) were assessed. These results revealed that the expression of COL2 was significantly upregulated from day 3, and elevated expressions was detected at day 5 and 7 (*p* < 0.05) (Fig. 5a). In addition, the expression of another cartilage-related transcription factor SOX9 also upregulated from day 5 (*p* < 0.05) (Fig. 5b). However, the cartilage matrix gene ACAN showed no significant changes at all the time points (*p* > 0.05) (Fig. 5c). Regarding the expression of COL1 gene, a downregulation was observed at day 5, however, it tended to upregulate at day 7 (Fig. 5d). With respect to stemness-related genes, we evaluated the changes of OCT-4 and Nanog expression. The expression of these two genes tend to decreased gradually with increasing culture time (Fig. 5e, f). Changes of Nanog expression showed statistically significant differences at day 7 (*p* < 0.05), while no significant difference was detected in OCT-4 expression at all the time point (*p* > 0.05) (Fig. 5e, f).

## Discussion

Acute articular cartilage damage is one of the most challenging clinical problems for orthopedic surgeons. To restore articular cartilage, biological cells-based regenerative strategies have been developed [7]. TSPCs, a type of MSC have been extensively used in tissue repair, however, restricted for tendon tissue repair [25–30]. The reason that it is not broadly used for other tissue repair is that its accessibility is harder than the widely used BMSCs and ASCs. In addition, donor site morbidity after autologous tendon tissue harvest is also concerned. It's worth noting that articular cartilage lesions usually occur in conjunction with other musculoskeletal injuries, such as ACL tears, and the standard surgical procedure is to reconstruct the torn ligament with autologous tendon autografts [1]. Thus, we hypothesize that autologous TSPCs can be isolated from a portion of the tendon autograft for cartilage repair. According to literature, TSPCs have been reported to show higher clonogenicity and proliferative capacity, and greater chondrogenic differentiation potential than BMSCs and ASCs [16]. Based on above, TSPCs might be a better cell source and has been overlooked as reparative cell source for cartilage regeneration.

The matrix surrounding the cells provides a unique microenvironment and plays a significant role in regulating chondrocyte differentiation and maintaining cartilage function in natural cartilage tissue [31–35]. In recent years, decellularized biomaterials derived from various tissues have been used in the regenerative medicine for different tissues and organs, including tendons, ligaments, blood vessels, skin, nerves, skeletal muscle, bladder, etc. [36–41]. In the current study, we used the previous described cartilage decellularization method to prepare DCM [5]. After obtaining fresh porcine cartilage, the cellular components were removed by repeat freeze–thaw cycles and chemical decellularization procedures. DAPI staining and HE staining showed that the nuclei were largely removed from the cartilage lacuna, leaving only the spaces where chondrocytes were previously present. Quantification data further confirmed that most of the DNA in the DCM was removed, with a concentration of 1.5 ng/mg after decellularization, which is well below the limit for medical implant devices (50 ng/mg) [42]. At the same time, the basic tissue structure and more than half of the GAGs and collagen were preserved, which are important components of the cartilage extracellular matrix. Chemical decellularization agents that remained in the DCM may be toxic. Live/dead staining and CCK-8 assay were performed to test the biocompatibility of the DCM. Live/dead staining showed that chemical toxicity was largely removed by the repeated washing steps during the decellularization procedures and hTSPCs could attach to and survive on it with high viability. In addition, CCK-8 assay indicated that the cell number of hTSPCs gradually increased with culture time. We also compare the proliferative activity of hTSPCs on the DCM and two-dimensional cell culture flask, and no significant difference was detected. This was consistent with results reported by previous studies [43].

Next, we investigated if hTSPCs would undergo chondrogenic differentiation in a chondrogenic microenvironment. After cultivation on the DCM, qPCR of several chondrogenic- and stemness-related genes were conducted. COL2, a typical cartilage matrix gene and SOX9, a chondrogenic transcription factor are linked to early cartilage formation. At day 5, both genes up-regulated, suggesting that DCM induced chondrogenesis of hTSPCs. A higher expression level of COL2 at day 7 is encouraging because collagen type 2 synthesis and assembly is generally regarded as a barrier in cartilage repair and formation [44]. Moreover, the stemness-related gene, NANOG tend to decrease gradually with increasing culture time, and showed significant down-regulation at day 7, indicating the differentiation of hTSPCs towards a mature cell type [45–47]. COL 1 is one of the highly expressed genes of TSPCs, whereas, it is also a dedifferentiation marker for chondrocytes. In the current study, a downregulation of COL 1 was observed at day 5, however, it tended to upregulate at day 7. It may attribute to the intrinsic property of TSPCs, or it may also relate to dedifferentiation of cells during 2D cell cultivation. Further in-depth molecular study should be performed to clarify this phenomenon. Based on these findings, the DCM appears to provide a suitable microenvironment for hTSPCs growth and chondrogenic differentiation. Other numerous studies also have reported that cartilage ECM-based biomaterials induced chondrogenic differentiation of MSCs, even though without the use of exogenous growth factors [5, 48]. The potential mechanism that the DCM enhances the chondrogenesis of hTSPCs may relate to the interaction of the cells with the natural chondrogenic niche. In addition, some growth factors or functional proteins retained in DCM may also play an important role in chondrogenic differentiation of hTSPCs. Our previous studies have revealed that certain amount of growth factors, such as TGFb, FGF, and IGF were detected within decellularized cartilage matrix [5]. Further studies are required to investigate the precise mechanism how the DCM determines the chondrogenesis of hTSPCs.

Clinically, patients undergo associated cartilage lesions with ligament injuries, autologous TSPCs can be isolated from a portion of the tendon autograft harvested for ligaments reconstruction. Next, the isolated TSPCs could be combined with biomaterials, such as cartilage-derived matrix for repairing the cartilage lesions. Even for the elderly group, the multipotency of TSPCs is not influenced by tendon ageing, and still have a strong chondrogenic differentiation potential [14]. Hence, this strategy may also be suitable for the elderly patients. Clinically, the application of ACI is strictly restricted by age, because the quality of autologous chondrocytes is majorly affected by aging. One of the limitations of the current study is that only one cell type, hTSPCs was evaluated, with no other MSCs included for comparison. Although the chondrogenic capacity of TSPCs has been documented to be higher than that of ASCs and BMSCs, this was not reflected in this experiment. However, this study is focused on the situation of associated articular cartilage lesions in conjunction with ligament injuries. In this cohort, autologous TSPCs can be isolated from a portion of the tendon autograft harvested for ligaments reconstruction. Thus, other MSCs types are not considered for application in promoting cartilage repair. It has been shown that chondrogenesis and cartilage tissue repair can be enhanced in vitro or/and in vivo by the deculturized cartilage matrix. However, there are differences in the microenvironment in vivo versus in vitro, especially the unfavorable microenvironment after trauma. Thus, animal experiments will be carried out in the future by implementing clinically relevant models od cartilage lesions in conjunction with ligament injuries.

## Conclusion

By decellularizing porcine cartilage, we obtained the DCM and provide a chondrogenic microenvironment for hTSPCs. The results indicated that the DCM supported hTSPCs attachment and proliferation with high biocompatibility. Moreover, the DCM microenvironment induced chondrogenic differentiation of hTSPCs, without the use of exogenous growth factors. There results indicated that TSPCs are promising reparative cell sources for promoting cartilage repair. Especially, in the cohort that articular cartilage lesions occur in conjunction with ligament injuries, autologous TSPCs can be isolated from a portion of the tendon autograft harvested for ligaments reconstruction.

## Data Availability

The datasets used and/or analyzed during the current study are available from the corresponding author on reasonable request.
